# The effect of childhood family adversity on adulthood depression among Chinese older migrant workers: gender differences in the mediating role of social-ecological systems

**DOI:** 10.1186/s12889-024-19397-7

**Published:** 2024-07-26

**Authors:** Xiaoyue Liu, Arlette J. Ngoubene-Atioky, Xudong Yang, Yuanping Deng, Jiayi Tang, Liujun Wu, Jiahui Huang, Yawen Zheng, Juan Fang, Amrita Kaur, Li Chen

**Affiliations:** 1 School of Mental Health, Wenzhou Medical University, Wenzhou, China; 2 Center for Psychology, Goucher College, Baltimore, MD USA; 3 Cixi Biomedical Research Institute, Wenzhou Medical University, Ningbo, China; 4 Normal College, Jimei University, Xiamen, China; 5 School of Psychology, Wenzhou-Kean University, Wenzhou, China; 6 The affiliated Wenzhou Kangning Hospital, Wenzhou Medical University, Wenzhou, China

**Keywords:** Social-ecological systems, Childhood family adversity, Depression, Older migrant workers, Gender differences

## Abstract

**Background:**

Older migrant workers (OMWs) in China face unique challenges rooted in their early life experiences, which increase their vulnerability to psychological and behavioral problems in adulthood. By utilizing the cumulative disadvantage model and the social-ecological systems theory, this study explored the effect of childhood family adversity on adulthood depression in the mediating roles of OMWs’ social-ecological microsystem and mesosystem and further examined gender differences in these associations.

**Methods:**

Data were collected from the China Health and Retirement Longitudinal Study (CHARLS), involving a sample of 4,309 OMWs aged 50 and above. The measures included the Center for Epidemiological Research Depression Scale, childhood family adversity, socioeconomic status, marital quality, and physical and cognitive health.

**Results:**

Childhood family adversity was positively associated with adulthood depression among OMWs. Social microsystem (physical and cognitive health) and mesosystem (marital quality and socioeconomic status) factors significantly mediated this relationship. Multi-group analysis revealed that the mediating effects of marital quality and socioeconomic status were stronger for female OMWs, while the mediating effects of physical and cognitive health were stronger for male OMWs.

**Conclusions:**

The findings suggest that childhood family adversity has a lasting impact on the mental health of OMWs, and that social-ecological systems factors play an important role in this relationship. The study also highlights the need for gender-specific interventions to address the mental health needs of OMWs.

## Background

China’s swift urbanization has attracted a large number of migrant workers from rural areas seeking improved employment opportunities and living standards in urban areas. These workers, without a permanent urban ‘hukou’, constitute over one-third of China’s labor force [1]. As the population ages, older migrant workers (OMWs) aged 50 and above have become a distinct group [2–4]. In 2022, 29.2% of rural-to-urban migrant workers in China were OMWs, marking a 6.8% increase since 2018 [5].

Despite their significant contributions to cities, OMWs encounter age-related challenges like reduced working capacity, lower educational levels, and limited training opportunities [6, 7]. The urban-rural dual system also restricts their access to social welfare services, leading to social exclusion and discrimination [8–10], and a higher likelihood of being employed in lower-paying, physically demanding, and high-risk industries [11]. Moreover, a significant number of OMWs have faced a multitude of early-life adversities, ranging from economic hardships such as poverty, to various forms of maltreatment like physical abuse and domestic violence [12]. These mentioned factors make OMWs highly susceptible to depression. Studies indicate that OMWs have a higher prevalence of depression (20–35%) than older urban workers (10–25%) [2, 13, 14]. Consequently, it is crucial to explore the risk factors contributing to depressive symptoms in OMWs and develop effective coping strategies.

The current study employs the cumulative disadvantage model of life course theory [15] and social-ecological systems [16] to establish a comprehensive framework that explores the potential mechanisms connecting childhood family adversities to adult depression among OMWs.

### Theoretical framework

Scholars have recently combined the life course perspective with the cumulative disadvantage theory to explain how early adverse experiences can increase an individual’s risk for health problems later in life [17–19]. Specifically, individuals who encounter greater adversity in early life are more prone to health challenges, difficulties in managing stress in adulthood, and facing additional disadvantages such as lower educational attainment [20], limited job opportunities [21], and lower socioeconomic status (SES) [22], all of which can contribute to mental and physical health problems [19, 23]. Therefore, this study posits that heightened childhood family adversity may elevate the susceptibility to OMWs’ depression in adulthood and exacerbate the accumulation of disadvantages across pivotal life stages.

Another theory that underpins this study is the social-ecological systems theory, it was first proposed by Bronfenbrenner (1979) and further developed by Charles Zastrow (1997) [16, 24]. Zastrow suggests that an individual’s mental health is impacted by three layers of social systems, including the microsystem (e.g., physical, psychological conditions), mesosystem (e.g., family, friends) and macrosystem (e.g., government, institution) [24, 25]. A microsystem is an apparently single individual in a social-ecological environment. It includes the biological and psychological aspects of a person. Mesosystem refers to the socioecological elements that directly interact with a person’s life development, including families, occupational groups or other social groups. Macrosystem is a social system larger than small groups, including cultures, communities, institutions and organizations. By integrating the cumulative disadvantage theory from the life course perspective, this study aims to evaluate the relationship between childhood family adversity and depression in OMWs, and explores how the microsystem (physical and cognitive health), mesosystem (marital quality and SES), and gender identification influence this relationship.

### Childhood family adversity and adulthood depression

Childhood family adversity encompasses early life stressors like family poverty, parental abuse, neglect, mental health issues, and poor parental relationships [19, 26]. Migrant workers often experience more family adversity in early life, increasing their risk of mental problems in adulthood [27–29]. For example, one cross-sectional study of 1,563 migrant workers found that childhood experiences of abuse may be a contributory factor to mental health problems, even suicidal behaviour in adulthood [30]. Furthermore, individuals with higher cumulative disadvantage early in life may have poorer physical health [31], lower cognitive reserve [32], lower marital quality [33] and lower SES [22] in adulthood, which may contribute to depression [19, 23]. In summary, childhood family adversity is associated with depression and may lead to poorer individual’s social-ecological systems in adulthood, further exacerbating depression.

### Physical and cognitive health in social-ecological microsystem as mediators

Based on the social-ecological systems theory, physical and cognitive health are crucial components of the social microsystem, significantly impacting an individual’s depression [34–36].

Poor physical health can hinder social interactions, foster social isolation [37] and trigger feelings of sadness and diminished self-esteem, which are common indicators of depression [38, 39]. Furthermore, the physical health of adults can be affected by childhood family adversity, which in turn can contribute to depression in adulthood [40]. The cognitive model of depression [41] also suggests that individuals with depression experience stable cognitive difficulties such as short attention span, memory deficits, negative thinking patterns, and cognitive distortions [42], impacting daily functioning and subjective quality of life. Additionally, cognitive functioning in adulthood may be influenced by childhood family adversity [43–46], potentially making older adults more susceptible to cognitive decline and dementia [32].

In summary, physical and cognitive health (microsystem) are crucial mediators in the pathway that links childhood family adversities to adulthood depression.

### Marital quality and SES in social-ecological mesosystem as mediators

Based on the social-ecological systems theory, marital quality and SES are considered as crucial components of the social mesosystem, and have a significant impact on an individual’s depression.

Marital quality is a critical component of family dynamics, evaluating the contentment and well-being of a spouse’s relationship [47]. Lower marital quality can restrict access to social support and heighten feelings of isolation, which can contribute to mental anguish and depression [48]. Studies have indicated that migrant workers with lower marital quality are more prone to experiencing symptoms of depression [49]. Following the principles of the Bowen family systems theory [50], past traumatic experiences can impede the ability to establish and sustain healthy intimate relationships, potentially leading to marital discord and dissatisfaction [33, 51].

SES is a measure of an individual’s or group’s economic and social position in relation to others, based on factors such as income, education and occupation [52]. OMWs frequently have low education levels and reside in peripheral urban areas, hindering their access to adequate socioeconomic welfare [53, 54]. Consequently, OMWs with lower SES may possess limited social capital, encounter greater social pressure, and experience higher levels of social exclusion, all of which can heighten their vulnerability to depression [55]. The SES individuals attain in adulthood can be influenced by adverse childhood experiences [56, 57].

In summary, marital quality and SES (mesosystem) serve as crucial mediators in the pathway that links childhood family adversities to the adulthood depression.

### Gender differences in the relationship between childhood family adversity and adulthood depression

According to social role theory [58] and Chinese culture, men are dominant in society, while women are often subordinate, particularly in rural areas [59–61]. Research has shown that gender role socialization can influence how individuals develop coping mechanisms and handle external challenges [62]. Therefore, gender is a crucial factor in exploring the connection between childhood family adversity, social microsystem (physical and cognitive health), social mesosystem (marital quality and SES), and depression. However, there is a research gap regarding gender differences among OMWs in this context.

### The present study

In summary, the existing body of research has primarily focused on examining the relationship between childhood adversity and depression, with limited attention given to the potential mediating role of social-ecological systems, particularly in the context of OMWs. There is also a gap in studies exploring gender differences in this correlation. By identifying these gaps in the literature, we aim to justify our research focus on understanding how social-ecological systems mediate the link between childhood family adversity and depression among OMWs in China, as well as investigating potential gender disparities in this context.

**Fig. 1:**
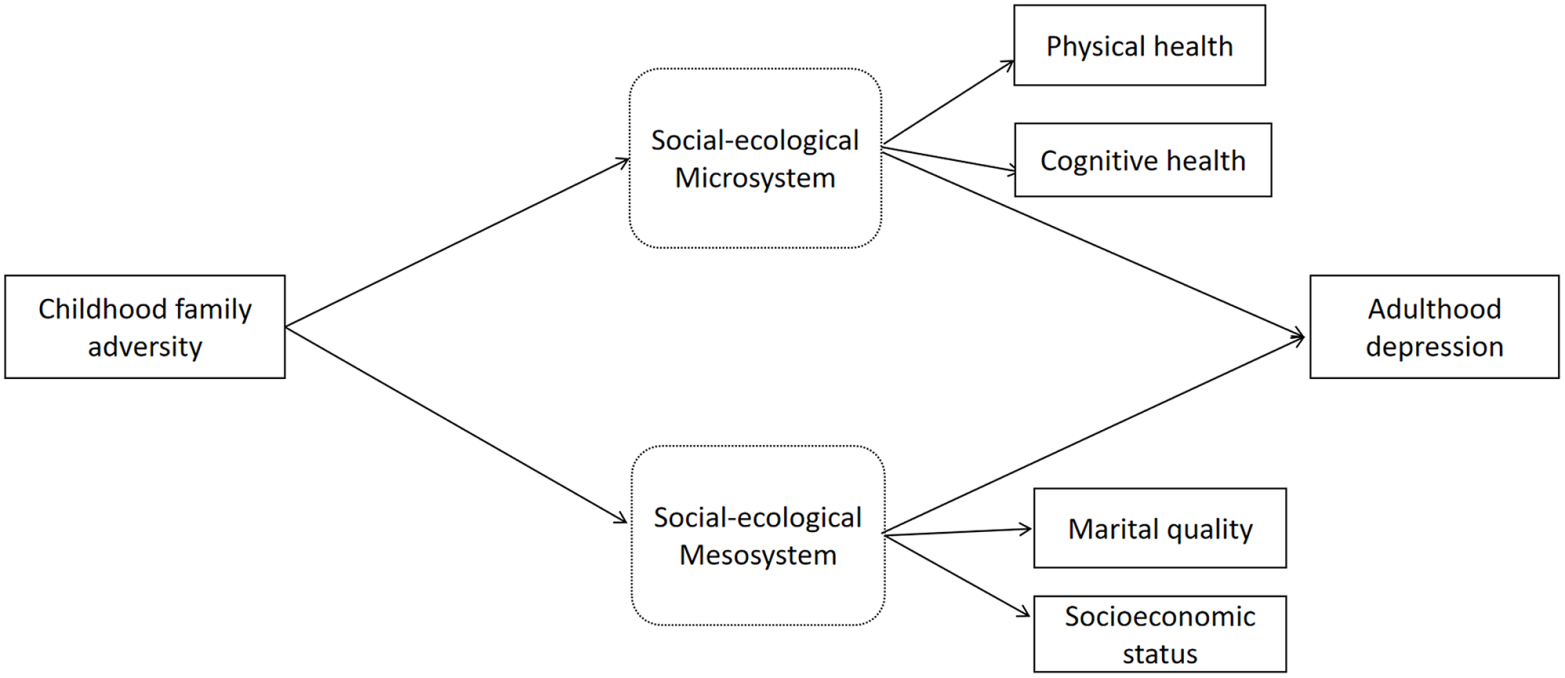
Model of the relationship between childhood family adversity and adulthood depression, mediated by social-ecological system

Four research hypotheses are proposed: [1] childhood family adversity significantly predicts depression in adulthood among OMWs; [2] an individual’s social microsystem (physical and cognitive health) mediates the relationship between childhood family adversity and depression among OMWs; [3] the social mesosystem (marital quality and SES) of OMWs mediates the link between childhood family adversity and depression outcomes; [4] there are gender differences in the association between childhood family adversity and depression among OMWs (see Fig. 1).

## Method

### Data and sampling

This study utilized data were derived from the 2018 waves of the China Health and Retirement Longitudinal Study (CHARLS), a nationally representative longitudinal survey of China’s middle-aged and older adults. It comprises a three-stage stratified probability proportionate to size sample. CHARLS covers 28 provinces, 150 counties or districts and 450 urban communities or villages across the country. A structured questionnaire was used to collect their health status and other relevant information via face-to-face interviews [63]. Previous studies described detailed information [64, 65] and are available at the CHARLS website (http://charls.pku.edu.cn/). Ethical approval was obtained from the Biomedical Ethics Review Committee of Peking University, and all participants provided written informed consent. As childhood family adversity was not evaluated in the 2018 wave, the data was retrieved from the 2014 wave and participants were matched one-to-one based on their unique identifiers. The sample consisted of individuals aged 50 and above, who were identified as married migrant workers with rural area household registration. These individuals had engaged in one or more non-farm jobs for at least three months in the past year, and their current residence was in cities and towns. Finally, 6,504 participants met the criteria for this study, and 2,195 were excluded due to missing data, leaving a final sample of 4,309. Independent sample t-tests were used to compare differences between the final included cases and the excluded cases, and it was found that only age and physical health variables had differences at the 0.05 level, but not at the 0.01 level of significance; the other variables had no differences between the study variables at the 0.05 level of significance. The missing data basically followed a completely random missing pattern. Among them, 2,616 (60.71%) were male and 1,693 (39.29%) were female, with a mean age of 58.24 (± 6.90), income 5.78 (± 4.49) and education 5.63 (± 3.33).

### Measurements

#### Childhood family adversity

The assessment of childhood family adversity was based on previous studies [18, 66, 67], specific situations prevalent in Chinese migrant workers’ families, and childhood adverse events experienced before the age of 17. The assessment considered eight factors, including parental physical abuse, parental emotional neglect, parental psychiatric disorder, parental marital discord, early parental death, parental separation, poverty and childhood food inadequacy [67, 68]. For example “Did your male guardian have abnormality of mind when you were young?”. Each item was scored in a binary of either 0 for no endorsement of such an experience and 1 for endorsement of this experience. Then, we combined scores on the eight childhood family adverse events to construct an index of childhood family adversity which ranged from 0 to 8. If a participant reported four or more adverse experiences, we assigned a value of 4. This decision was made because the proportion of participants with five or more adverse experiences was very small. Higher scores indicated a greater number of childhood family adversity. These tests have been widely used and validated for measuring childhood adversity in Chinese adults in previous studies [67, 69]. In our study, the Cronbach’s alpha of childhood family adversity is 0.871.

#### Physical health and cognitive health in social-ecological microsystem

Physical health was evaluated using the item “What do you think about your physical health?” from the CHARLS study. Participants rated their current physical health on a Likert-type scale ranging from 1 (very good) to 5 (very bad), with higher scores indicating poorer physical health.

Cognitive health was measured using an adapted Chinese version of the Mini-Mental Status Examination (MMSE) [70], which is used to measure cognitive function in the US Health and Retirement Study (HRS). Internal consistency appears to be moderate with Cronbach’s alpha scores reported between 0.6 and 0.9 [70–73]. Test-retest reliability has been examined in several studies and in those where re-examination took place within 24-h reliability by Pearson correlation was usually above 0.85 [74, 75]. It focused on two areas of cognition, episodic memory and mental intactness [76, 77]. To measure episodic memory, interviewers read a list consisting of 10 Chinese words and asked participants to repeat as many words as she/he could remember (immediate recall), and to recall this word list five minutes later (delayed recall) [78, 79]. The final score of episodic memory was calculated as the average of immediate recall and delayed recall, ranging from 0 to 10. The mental status component consisted of time orientation, numerical ability and picture drawing. The measurement of time orientation required participants to recall today’s date (year, month, day), the day of the week and current season. Numerical ability required participants to attempt a serial subtraction of 7 starting from 100 (up to 5 times), and the score of this part would be reduced by half if one used paper, pencil or other aid when completing the number subtraction. Scores of these two parts equal to the number of correct answers. When measuring ability of picture drawing, interviewers showed a picture of two pentagons overlapped to participants and asked them to draw that picture on a piece of paper. Participants who successfully reproduced the picture received 1 point, and those who failed to do so received a score of 0 [80]. The total score of mental status was the summation of score from time orientation, numerical ability and picture drawing, ranging from 0 to 11. In order to assess the overall cognitive functions, we defined global cognition as the total score of episodic memory and mental status on a scale from 0 to 21, with a higher score indicating superior cognitive functions. These tests have been widely used and validated for measuring cognitive health in older Chinese people and in previous studies [81, 82].

#### Marital quality and SES in social-ecological mesosystem

For marital quality, participants responded to the question, “Are you satisfied with your marriage?”, which was scored from 1 to 5 to represent very satisfactory to very unsatisfactory. Higher scores indicated a lower marital quality.

Based on previous studies [83–85], SES in our study was evaluated using three measures: education level, individual annual income, and occupation. For education level, participants responded to the question, “What is your education level?”, which was scored from 1 to 8 to represent illiterate to bachelor’s degree. For individual annual income, participants were asked to fill in the total amount of their income in Yuan for the past year. For the occupation section, participants were asked to fill in their occupations. Then, we used the 10-class EGP class schema [86] to classify occupation into 10 types in our study. Utilizing principal component analysis, a composite score of SES was constructed. The KMO value was 0.6, the Bartlett spherical test had a χ2 value of 264.19 (*P* < 0.001), indicating that it was suitable for factor analysis. Only the first principal component that had an eigenvalue greater than one was extracted. The variable SES was calculated by using the coefficients of three indicators in the factor score coefficient matrix, with the maximum and minimum scores ranging from 6.26 to − 1.32. Higher scores were indicative of higher SES. These tests have been widely used and validated for measuring SES in Chinese older adults in previous studies [87, 88].

#### Depression

The simplified version of Center for Epidemiological Research Depression Scale (CES-D) was used to measure depression [89]. The simplified version of CES-D consists of 10 multiple-choice items, and participants were asked about their feelings for 10 aspects during the last week, such as feeling bothered, having trouble concentrating and feeling depressed, with options including: almost none (less than one day), sometimes (1–2 days), often (3–4 days), most of the time (5–7 days). The corresponding score of each option is 0, 1, 2 and 3. The higher the total score is, the more severely depressed the participants are. The scale had exhibited good internal consistent reliability (Cronbach’s alpha = 0.813) [90, 91]. In the Chinese context, CESD-10 has been extensively utilized and validated as a valuable and dependable tool for identifying the risk of depression among the older population, aiding in further diagnosis [92, 93]. The Cronbach’s alpha for the CESD-10 in our study was 0.788.

#### Statistic analysis

Statistical analyses were performed using SPSS 25.0 and R 4.1.2. Childhood family adversity was analyzed using frequency statistics, while depression, SES, marital quality, physical health, and cognitive health were examined using variance analysis among OMWs with different levels of adversity. Variable correlations were assessed using Pearson’s correlation. A structural equation model (SEM) with 5000 bootstraps and a 95% confidence interval was employed to investigate mediation effects. Additionally, a multi-group SEM was utilized to explore gender differences as a potential moderator.

The analysis involved three steps: Model 1 with freely estimated parameters, Model 2 with constrained factor loading of latent variables, and Model 3 with equal path coefficients across samples. Gender, age, number of children, and chronic disease were controlled for in all models.

The model fit was assessed in multiple ways [94]. The χ^2^ test was first used to assess the fit of the model. According to previous research [95], χ^2^/df values less than 5 are a criterion for a robust fit. However, since the χ^2^ test depends on the sample size, the larger sample size of the current study may have compromised the fit. Therefore, the goodness-of-fit indices, specifically, the Comparative Fit Index (CFI), the Normal of Fit Index (NFI), and the Root Mean Square Approximation Error (RMSEA), were also assessed. The CFI and NFI values being higher than 0.90 indicated great fit while RMSEA values being lower than 0.07 also denoted good fit of the model [96–98].

## Results

### Preliminary analysis

The frequency, percentage and score for each variable of family adversity in childhood are shown in Table 1. Of the 4,309 OMWs, 45% experienced at least one type of family adversity, with 28% experiencing one type, 11% experiencing two types, and 5% experiencing three or more types. Analysis of variance revealed significant differences among OMWs who had experienced different levels of adversity in terms of depression, marital quality, SES, physical health, and cognitive health. OMWs who experienced more childhood family adversities had higher depression levels, lower marital quality, lower SES, and poorer physical and cognitive health than those with fewer childhood family adversities. Pearson’s correlations among the study variables are provided in Table 2. As expected, childhood family adversity was significantly correlated with physical health, cognitive health, marital quality, SES and depression (*ps* < 0.001). Depression was significantly correlated with physical health, cognitive health, SES and marital quality (*ps* < 0.001).

**Table 1:** Cumulative childhood family adversity of OMWs and its distribution in other variables

Cumulative family adversity in childhood	*N*(%)		Depression	Social-ecological Microsystem		Social-ecological mesosystem	
				Cognitive health	Physical health	Marital quality	SES
0①	2377	55.164	17.066 ± 5.776	12.113 ± 4.083	2.692 ± 1.014	2.486 ± 0.745	0.157 ± 0.756
1②	1233	28.615	17.822 ± 5.835	11.608 ± 4.230	2.816 ± 1.017	2.547 ± 0.785	0.050 ± 0.730
2③	480	11.139	18.750 ± 6.231	11.257 ± 4.369	2.892 ± 1.036	2.585 ± 0.768	-0.022 ± 0.723
3④	161	3.736	19.925 ± 7.150	11.422 ± 4.031	3.106 ± 1.010	2.702 ± 0.948	-0.044 ± 0.776
≥4⑤	58	1.346	21.172 ± 7.260	9.621 ± 4.423	3.259 ± 0.983	2.690 ± 0.922	-0.327 ± 0.695
*F*			21.234^**^	10.367^**^	13.524^**^	5.263^**^	14.258^**^
Post-hoc			① < ② < ③ < ④, ⑤	① > ②, ③ ,④, ⑤ ②, ③, ④ > ⑤	① < ②, ③, ④, ⑤ ②, ③ < ④, ⑤	① < ②, ③, ④, ⑤ ② < ④	① > ②, ③, ④, ⑤ ②, ③, ④ > ⑤

Note: SES - Socioeconomic status; ^*^ < 0.05, ^**^ < 0.01, ^***^ < 0.001

**Table 2:** Correlations and descriptive statistics for the main study variables (*N* = 4309)

Variables	1	2	3	4	5	6	7	8	9	10
1. **Age**	1				.					
**2. Gender**	− 0.207^***^	1								
**3. Number of children**	0.375^***^	− 0.063^***^	1							
**4. Chronic disease**	0.08^**^	− 0.023	0.040^**^	1						
**5. Childhood family adversity**	0.056^**^	− 0.036^*^	0.037^*^	0.059^***^	1					
**6. Cognitive health**	− 0.208^***^	− 0.038^*^	− 0.105^***^	− 0.015	− 0.091^***^	1				
**7. Physical health**	0.095^***^	0.024	0.074^***^	0.274^***^	0.110^***^	− 0.125^***^	1			
**8. Marital quality**	− 0.003	0.200^***^	0.014	0.046^**^	0.068^***^	161^***^	0.179^***^	1		
**9. SES**	− 0.170^***^	− 0.153^***^	− 0.149^***^	− 0.040^**^	− 0.111^***^	0.373^***^	− 0.125^***^	− 0.021	1	
**10. Depression**	0.023	0.142^***^	0.063^***^	0.162^***^	0.138^**^	− 0.179^***^	0.370^***^	0.265^***^	− 0.182^***^	1
***M***	58.242	0.607	2.203	0.393	0.675	0.092	2.770	2.525	11.814	17.632
***SD***	6.900	0.488	1.007	0.489	0.910	0.749	1.023	0.772	4.179	5.978

Note: SES - Socioeconomic status; ^*^ < 0.05, ^**^ < 0.01, ^***^ < 0.001; gender: 1- male, 0 - female; Chronic disease: 1- with, 0 - without

### Testing the mediating effect of social microsystem and mesosystem

**Fig. 2:**
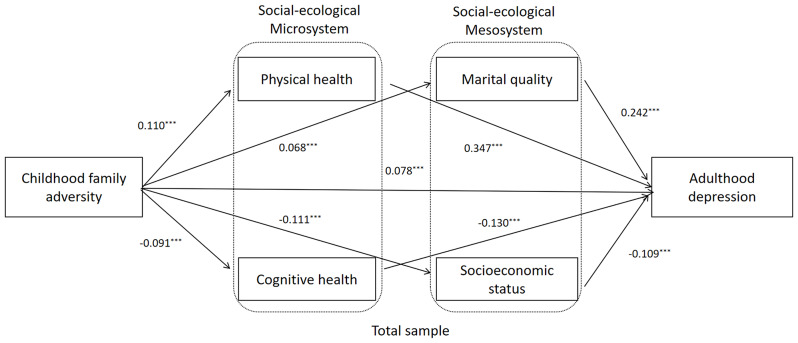
Mediation model from childhood family adversity to adulthood depression of OMWs. Note Standardized structural model (Total sample). OMWs: Older Migrant workers. * < 0.05, ** < 0.01, *** < 0.001. Gender, age, number of children, and chronic disease were controlled in model

SEM was used to examine the mediating role of microsystem (physical and cognitive health) and mesosystem (marital quality and SES) between childhood family adversity and adulthood depression. Figure 2 presents the standardized coefficient of the parallel mediation model which revealed optimal fit indices (χ^2^ = 714.783, *df* = 37, χ^2^/*df* = 19.302, *p* < 0.001, RMSEA = 0.065, CFI = 0.926, NFI = 0.922, TLI = 0.900), childhood family adversity (*β* = 0.07, *p* < 0.001), physical health (*β* = 0.347, *p* < 0.001), cognitive health (*β* = − 0.13, *p* < 0.001), marital quality (*β* = 0.24, *p* < 0.001), and SES (*β* = − 0.10, *p* < 0.001) significantly predicted adulthood depression.

The mediating effects of physical health, cognitive health, marital quality, and SES were estimated using bootstrap analysis, as presented in Table 3. The 95% confidence interval for the standardized indirect effect did not include zero, indicating significant mediating effects. Specifically, childhood family adversity had a significant direct effect on depression level (effect = 0.07, *p* < 0.05, CI [0.04, 0.10]). Meanwhile, childhood family adversity had a significant indirect effect on depression via physical health (effect = 0.03, *p* < 0.01, CI [0.030, 0.049]), cognitive health (effect = 0.01, *p* < 0.05, CI [0.01, 0.01]), marital quality (effect = 0.01, *p* < 0.01, CI [0.01, 0.02]), and SES (effect = 0.01, *p* < 0.01, CI [0.01, 0.02]).

**Table 3:** The bootstrap confidence interval and effect size of the mediation model

Effects	Total example			Male example			Female example		
	Estimate	95%CI	*P*	Estimate	95%CI	*P*	Estimate	95%CI	*P*
**Direct effects**									
Gender → Depression	− 0.086	[-0.057, − 0.119]	0.010	-	-	-	-	-	-
Age → Depression	− 0.055	[-0.085, − 0.028]	0.010	− 0.057	[-0.099, − 0.019]	0.024	− 0.045	[-0.089, 0.003]	0.128
Number of children → Depression	0.039	[0.008, 0.066]	0.029	0.038	[-0.004, 0.077]	0.149	0.040	[-0.011, 0.089]	0.183
Chronic disease → Depression	0.082	[0.052, 0.111]	0.010	0.064	[0.025, 0.106]	0.010	0.106	[0.065, 0.146]	0.010
Childhood family adversity → Depression	0.078	[0.045, 0.105]	0.013	0.087	[0.058, 0.134]	0.003	0.078	[0.037, 0.117]	0.007
**Indirect effects**									
Childhood family adversity → Cognitive health - Depression	0.012	[0.007, 0.015]	0.016	0.009	[0.004, 0.015]	0.009	0.016	[0.009, 0.027]	0.008
Childhood family adversity → Physical health → Depression	0.038	[0.030, 0.049]	0.007	0.036	[0.025, 0.049]	0.005	0.044	[0.028, 0.058]	0.014
Childhood family adversity → SES - Depression	0.012	[0.011, 0.024]	0.005	0.010	[0.005, 0.015]	0.012	0.014	[0.008, 0.024]	0.008
Childhood family adversity →Marital quality → Depression	0.017	[0.011, 0.024]	0.005	0.010	[0.004, 0.016]	0.017	0.030	[0.015, 0.04]	0.014

Note: SES - Socioeconomic status. Gender, age, number of children, and chronic disease were controlled in model

### Multi-group analysis for the male OMWs and female OMWs

Group differences between male and female OMWs were analyzed using multi-group analysis in SEM. Table 4 displays three models: an unconstrained baseline model (Model 1), a model with constrained factor loadings across groups (Model 2), and a model with constrained factor loadings, item intercepts, and latent means across groups (Model 3). Model fit indices indicated that all three models fit well (CFIs > 0.90, NFIs > 0.89, RMSEAs < 0.07). However, Chi-square tests showed that the constrained Model 3 significantly differed from the unconstrained Model 1 (model 3: Δχ^2^ = 61.97, *p* < 0.001), suggesting that at least one path coefficient varied between male and female OMWs.

**Table 4:** The fitting index of multiple-group analysis model

	χ2	df	χ2/ df	Δχ2	*P*	RMSEA	CFI	NFI	AIC	ECVI
**The unconstrained baseline model**	2348.852	198	11.868	-	-	0.050	0.911	0.904	2488.975	0.478
**The measurement weights constrained model**	2363.563	207	11.416	14.711	0.099	0.049	0.910	0.903	2510.813	0.483
**The structural weights constrained model**	2410.829	227	10.616	61.977	0.000	0.048	0.903	0.894	2653.947	0.515

**Fig. 3:**
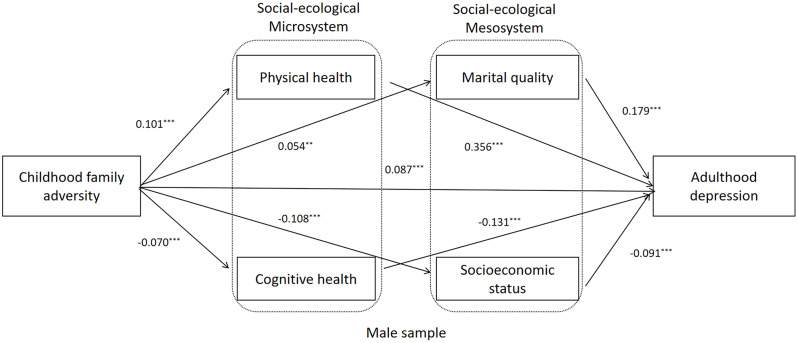
Mediation model from childhood family adversity to adulthood depression of male OMWs. Note: Standardized structural model (Male sample). OMWs: Older Migrant workers.^*^ < 0.05, ^**^< 0.01, ^***^ < 0.001. Age, number of children, and chronic disease were controlled in model

**Fig. 4:**
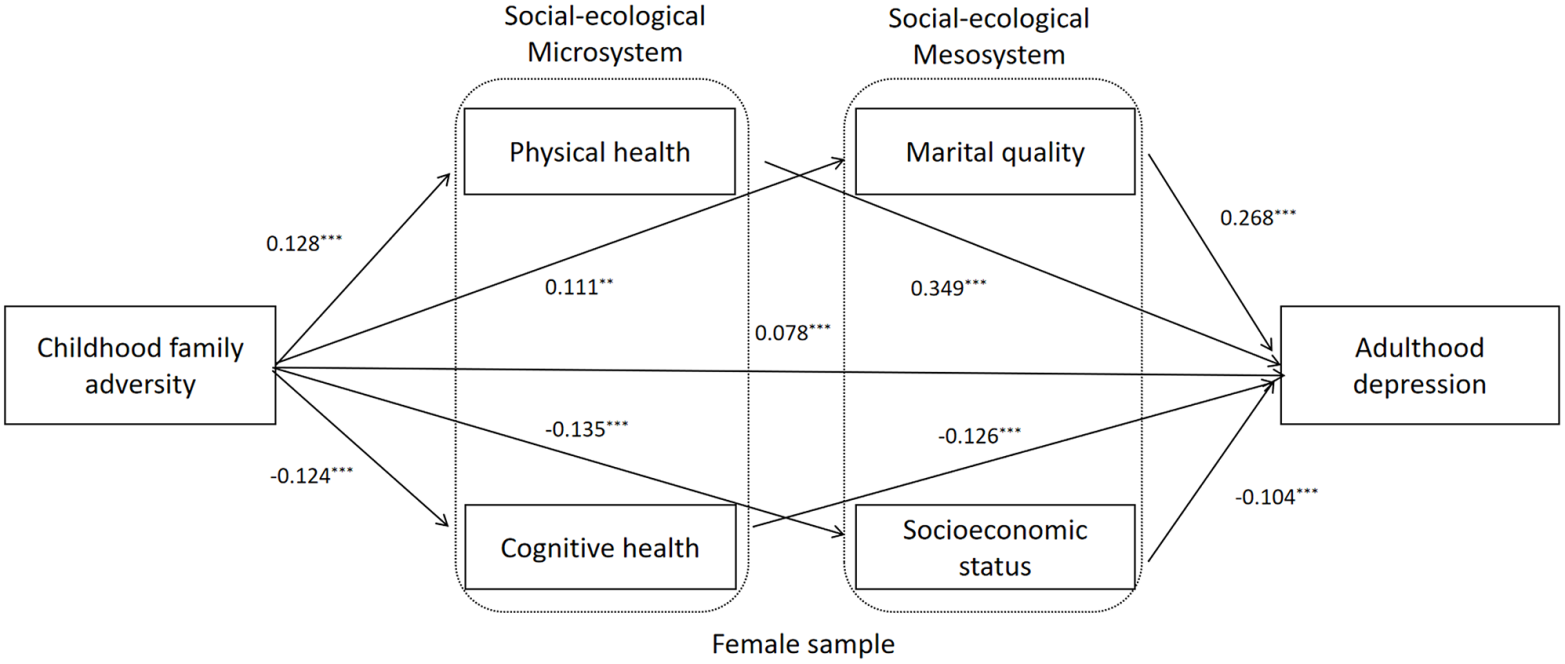
Mediation model from childhood family adversity to adulthood depression of female OMWs. Note: Standardized structural model (Female sample). OMWs: Older Migrant workers. ^*^ < 0.05, ^**^ < 0.01, ^***^ < 0.001. Age, number of children, and chronic disease were controlled in model

To further investigate the differences between male and female OMWs, Critical Ratios of Differences (CRDs) were used to examine the path coefficients in the multiple-group analysis model. Figures 3 and 4, and Table 5 present the results. The path coefficients from childhood family adversity to marital quality and marital quality to depression were significantly lower for male OMWs (*β* = 0.05, *p* < 0.001; *β* = 0.17, *p* < 0.001) than for female OMWs (*β* = 0.11, *p* < 0.001, CRD = − 2.33, *p* < 0.05; *β* = 0.26, *p* < 0.001, CRD = − 2.89, *p* < 0.01). Additionally, the path coefficients from childhood family adversity to cognitive health and physical health to depression were significantly higher for male OMWs (*β* = -0.07, *p* < 0.001; *β* = 0.35, *p* < 0.001) than for female OMWs (*β* = − 0.12, *p* < 0.001, CRD = 2.23, *p* < 0.05; *β* = 0.34, *p* < 0.001, CRD = 2.03, *p* < 0.05).

**Table 5:** The critical ratios of path coefficient in the multiple-group analysis model

Path	Male	Female	CRD
Childhood family adversity → Depression	0.087^***^	0.078^**^	0.367
Childhood family adversity → Physical healthy	0.101^***^	0.128^***^	− 0.973
Childhood family adversity → Cognitive health	− 0.070^***^	− 0.124^***^	2.237^*^
Childhood family adversity → Marital quality	0.054^**^	0.111^***^	-2.339^*^
Childhood family adversity → SES	− 0.108^***^	− 0.135^***^	0.919
Physical healthy → Depression	0.356^***^	0.349^***^	2.037^*^
Cognitive health → Depression	− 0.131^***^	− 0.126^***^	− 0.152
Marital quality → Depression	0.179^***^	0.268^***^	-2.896^**^
SES → Depression	− 0.091^***^	− 0.104^***^	1.045

Note: SES - Socioeconomic status; ^*^ < 0.05, ^**^ < 0.01, ^***^ < 0.001

## Discussion

This study investigated the mediating role of an individual’s social microsystem (physical and cognitive health) and mesosystem (marital quality and SES) in the relationship between childhood family adversity and adult depression. Additionally, it examined gender differences in this relationship among OMWs.

### Childhood family adversity and adulthood depression

Our study discovered a direct correlation between the number of family adversities experienced during childhood and the levels of depression in OMWs. This aligns with the cumulative risk view, indicating that the impact of facing multiple adversities simultaneously is more harmful than facing a single adversity alone [99]. Furthermore, our findings also demonstrated a significant positive relationship between childhood family adversity and depression, supporting our initial hypothesis and consistent with previous studies linking childhood family adversity to a higher susceptibility to mental health disorders [19, 26]. Therefore, taking measures to prevent such experiences can be beneficial in reducing the prevalence of depression among OMWs.

### The mediating effects of social microsystem and mesosystem

Our study revealed that social microsystem (physical health and cognitive health) and mesosystem (marital quality and SES) of OMWs mediated the relationship between experienced childhood family adversity and later life depression outcome. Thus, the findings in this study validate hypothesis 2 and 3.

Childhood family adversity, such as poverty and conflict, can have a detrimental impact on the physical and cognitive health of Chinese migrant laborers due to the critical period of childhood development. Research shows that experiencing adversity in childhood is associated with a higher likelihood of engaging in risky health behaviors later in life, like smoking and alcohol dependence, which can significantly harm physical health [44, 45]. Furthermore, early exposure to adversity has the most profound and enduring negative effects on cognitive outcomes, influencing cognitive abilities in adulthood [100]. Additionally, individuals who experience poor physical and cognitive health may be at a higher risk of developing mental health problems (depression) [101].

The results show that childhood family adversity can impact an individual’s mesosystem, including marital quality and SES. Migrant laborers may experience social isolation, discrimination and stigmatization, which can affect their economic opportunities, social competence, self-beliefs and social status [102]. Childhood family adversity can shape individual’s developmental trajectory and available resources, leading to limited educational and occupational opportunities and a decreased likelihood of achieving high SES. Consequently, this may lead to reduced social capital, increased pressure, lowered core self-evaluation, social exclusion, and heightened risk of depression among migrant workers. According to Bowen family systems theory, traumatic experiences during early development may compromise an individual’s ability to initiate and maintain intimate relationships in adulthood, resulting in enduring negative effects on their intimate relationships [33, 50]. These difficulties may stem from the negative effects of childhood family adversity on interpersonal skills, emotional regulation, and attachment patterns. Furthermore, a discordant marriage can undermine the support resources available to an individual, such as spousal support, thereby exacerbating psychological stress and elevating the risk of depression [48].

Therefore, as the cumulative disadvantage theory and social-ecological systems theory suggests childhood family adversity can have a cascading effect on individuals’ microsystem and mesosystem, leading to depression.

### Gender differences in the relationship between childhood family adversity and adulthood depression

The study suggests that female OMWs may face greater vulnerability than males to the negative effects of childhood family adversity on marital quality. Furthermore, the relationship between marital quality and depression appears to be more significant for female OMWs. Conversely, the association between childhood family adversity and cognitive health, physical health, and depression is more prominent in male OMWs.

Female migrant workers in conservative rural Chinese environments may have experienced childhood adversity due to their gender roles. They are more likely to contemplate and share their early life experiences, which can impact their marital quality in adulthood. It is also noted that marital quality is known to be inversely associated with psychological distress, and this association is typically stronger among women than men [103]. These differences may be attributed to the differential impact of gender role socialization and the varying physiological and psychological responses to stress between genders. Traditionally, Chinese culture has placed a greater emphasis on male dominance and female subservience [104], resulting in women experiencing more stress compared to men. Moreover, women in China are often expected to fulfill multiple roles, including being a wife, mother, and caregiver, which can further exacerbate the effects of childhood family adversity on their marital quality and depression.

Additionally, our study found that childhood family adversity may have a greater impact on men’s cognitive health. We speculate that this may be due to the influence of social roles, which require men to be more involved in social competition, memory and problem-solving skills, whereas women are more sheltered and do not require many cognitive skills. Therefore, more childhood adversity may have a greater impact on men’s cognitive abilities than women’s. Furthermore, traditional Chinese cultural attribution of masculinity to strong body, career success, perseverance, invulnerability, physical strength, as well as independent, brave, decisive also explain our finding [105]. It is known that such physically laborious work may be carried out more by male migrant workers than female migrant workers [106]. Thus, pressure to adhere to cultural attribution of masculinity and laborious work conditions may explain male migrant workers’ susceptibility to the effects of physical health on depression.

## Conclusion and limitations

The study is limited by its cross-sectional design and reliance on secondary data, preventing the establishment of a causal relationship between childhood family adversity and depression. Future research should consider longitudinal or panel data to investigate causality. Additionally, the exclusion of unmarried OMWs may impact the generalizability of the findings. Another limitation is the self-reported nature of childhood family adversity data, which may introduce recall bias. To address this, future studies could employ multiple assessment methods, gather data at various time points, and utilize objective markers of adversity when possible.

This study, despite its limitations, is the first to delve into the potential mechanisms of depression among a large sample of OMWs from a life course perspective, utilizing a cumulative disadvantage hypothesis. The research reveals that the social-ecological microsystem (physical and cognitive health) and mesosystem (marital quality and socioeconomic status) of OMWs act as mediators between childhood family adversity and depression. The implications of the findings are threefold: firstly, there is a need for proactive risk assessment and ecologically-grounded mental health interventions for OMWs who have experienced early adversity. Secondly, protective strategies that target the alleviation of depression within OMWs’ social microsystem and mesosystem should be explored, including counseling, enhancing communication skills, ensuring access to health insurance, and promoting healthy lifestyle habits. Thirdly, interventions should take into account gender differences in depression outcomes, prioritizing marital quality for female migrant workers and physical health awareness for male migrant workers. Additionally, preventive measures should be implemented to raise awareness and reduce the impact of adverse events on the children of migrant workers. Ultimately, the study aims to draw attention to the importance of ecologically grounded health attributes of OMWs.

## Data Availability

The data that support the findings of this study are openly available in China Health and Retirement Longitudinal Study (CHARLS) at http://charls.pku.edu.cn/.

## Abbreviations

OMWs: Older migrant workers
CHARLS: China health and retirement longitudinal Study
SES: Socioconomic status
NNSE: Mini-mental status examination
HRS: Health and retirement study
CES-D: Center for epidemiological research depression scale
SEM: Structural equation model
CFI: Comparative fit index
NFI: Normal fit index
RMSEA: Root mean square approximation error
CRD: Critical ratios of differences
